# Nonlinear relationship between Silver Carp density and their eDNA concentration in a large river

**DOI:** 10.1371/journal.pone.0218823

**Published:** 2019-06-26

**Authors:** David P. Coulter, Ping Wang, Alison A. Coulter, Grace E. Van Susteren, Jessica J. Eichmiller, James E. Garvey, Peter W. Sorensen

**Affiliations:** 1 Center for Fisheries, Aquaculture, and Aquatic Sciences, Department of Zoology, Southern Illinois University, Carbondale, Illinois, United States of America; 2 BioTechnology Institute, University of Minnesota, Twin Cities, St. Paul, Minnesota, United States of America; 3 Department of Fisheries, Wildlife, and Conservation Biology, University of Minnesota, St. Paul, St. Paul, Minnesota, United States of America; National Oceanic and Atmospheric Administration, UNITED STATES

## Abstract

Although environmental DNA (eDNA) is increasingly being used to survey for the presence of rare and/or invasive fishes in aquatic systems, the utility of this technique has been limited by a poor understanding of whether and how eDNA concentrations relate to fish density, especially in rivers. We conducted a field study to systematically test whether the eDNA released by a model invasive fish, Silver Carp (*Hypophthalmichthys molitrix*), was related to the density of this species in a large river. We quantified fish density throughout the 460 km long Illinois River using hydroacoustic surveys at 23 sites while concurrently collecting 192 surface water samples for eDNA analysis. We found that Silver Carp numerical density and biomass density were positively and non-linearly related to eDNA concentration and detection rate. Both eDNA concentration (copy number) and detection rate increased rapidly as Silver Carp density increased but plateaued at moderate densities. These relationships could prove useful for estimating Silver Carp relative abundance in newly invaded locations where population numbers are low to moderate. Future studies should explore the causes of this nonlinear relationship as it would ultimately benefit aquatic species monitoring and management programs.

## Introduction

The ability to efficiently determine the presence of rare and/or invasive fishes and then assess either their abundance or biomass is requisite to developing management strategies for these species [1]. Reliance on traditional capture gears can be time-consuming, expensive, produce biases toward certain species or habitats, and may at times be ineffective [1–4]. Further, these gears may also cause stress, injury, or mortalities [5]. Because of the ease with which water can be sampled and the extreme sensitivity and specificity of qPCR, detection of the DNA released by organisms (environmental or eDNA) is now commonly being use to survey for rare [6–8] and/or invasive species [1,9–11].

While many studies have now shown that eDNA can be used to confirm the presence of fishes as part of surveillance programs (e.g., [1,8,12–14]), studies that address eDNA quantification have had mixed results. Many factors including water chemistry, temperature, species identity, and fish behavior influence fish eDNA-density relationships, and in large bodies of water these issues become more complex [15]. Nevertheless, linear relationships have generally been described between fish biomass and eDNA in small aquaria [16], ponds [17], and streams [18] (although this was not the case in one fast-flowing stream [19]). In contrast, nonlinear relationships have generally been described in lakes [20,21], although no apparent relationship was noted on one occasion that examined a set of large lakes [22]. In addition, one study systematically examined the relationship between eDNA concentration and fish abundance in a river and reported both nonlinear and linear relationships, depending on season and water temperature [23]. Finally, positive correlations were recently noted in tributaries of a large estuary [24]. It seems that these relationships become more complicated and less certain in larger water bodies. Accurately assessing fish abundance and distribution is likely a significant contributing challenge in large bodies of water, especially rivers [2,25], further complicating researchers’ ability to define eDNA-density relationships.

Quantifying relationships between eDNA concentration and density are of particular importance for managing invasive bigheaded (Asian) carps, *Hypophthalmichthys* species, in the large rivers they have invaded. Bigheaded carps were introduced to southern portions of the Mississippi River, USA watershed in the 1970s and now threaten to invade the Laurentian Great Lakes via the Illinois River, as well as the headwaters of the Mississippi River. These fishes threaten aquatic food web pathways [26,27], negatively affect commercially- and recreationally-harvested native fishes [28,29], dominate fish communities [30], and negatively affect recreation [31]. Intensive management and control efforts are being directed against these species, including the use of eDNA detection rates as a surveillance tool in uninvaded areas [32]. However, whether bigheaded carp density might also be assessed by eDNA concentration has seemingly not yet been examined.

This study sought to quantify relationships between eDNA concentration and density of one of the bigheaded carp species, Silver Carp (*H*. *molitrix*), throughout the 460 km long Illinois River, USA, a river important to the invasion ecology of this species. To determine quantitative relationships between fish density and eDNA concentration, we sampled water (eDNA) and fish density across a gradient of Silver Carp abundances in the Illinois River [30,33]. We then related Silver Carp eDNA to density estimates obtained from mobile hydroacoustic surveys. Our objective was to determine if there was a statistical relationship between Silver Carp density and eDNA concentration and/or detection rate.

## Methods

### Study sites

**Fig 1:**
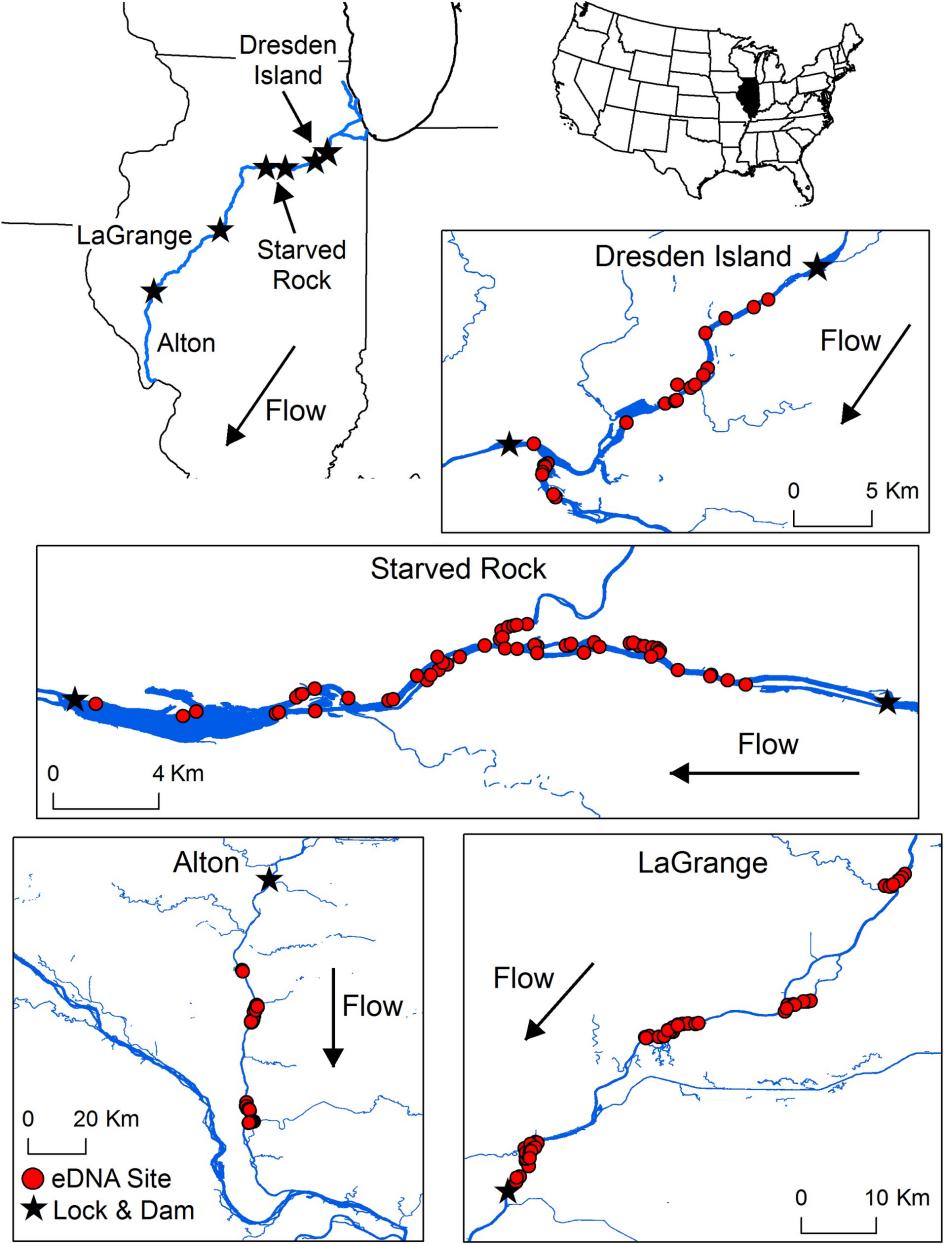
Locations (N = 192) across four reaches (Dresden Island, Starved Rock, LaGrange, Alton) of the Illinois River, USA where surface water samples were collected (eDNA Site) to assess Silver Carp eDNA concentrations. Simultaneous mobile hydroacoustic surveys were also conducted across all eDNA collection sites to quantify Silver Carp densities (survey paths not displayed).

Adult Silver Carp are currently located in the six reaches of the Illinois River, USA (Fig 1; 38.9686 N, -90.4681 W to 41.5022 N, -88.1047 W). The size of this river varies but its width is typically ≥ 150 m and thalweg depth is at least 2.7 m. We estimated Silver Carp densities and collected eDNA samples from 23 sites across four reaches where Silver Carp were present in the Illinois River from 13-Oct to 20-Oct in 2016 (Fig 1). Autumn sampling ensured spawning was not occurring [34] which might affect eDNA concentration due to gamete release [15,35]. Throughout the river, we sampled main channel, side channel, tributary, and backwater embayment habitats in the Dresden Island, Starved Rock, LaGrange, and Alton reaches. We started sampling in the most upstream reach and progressed downstream, as Silver Carp abundance increased from upstream to downstream reaches [30,33,36].

### Estimating silver carp density

All research followed the protocol approved by Southern Illinois University-Carbondale’s Institutional Animal Care and Use Committee (protocol number 17–003). Fish sampling followed established and validated protocols for hydroacoustic sampling [30,36,37] and had two components: 1) assessing fish species composition and sizes from physical catch data and 2) hydroacoustic sampling to quantify fish density. We used hydroacoustic sampling to evaluate Silver Carp density because it allowed us to sample across large geographic distances and a diversity of habitats and depths with the same gear (this can be difficult with other approaches in large rivers; [2]). Low water clarity prohibited visual counting of fish [23].

Physical catch data were collected just prior (Sept, 2016) to hydroacoustic and eDNA sampling (Oct, 2016) and were used in the analysis of hydroacoustic data. Fish sampling used a combination of pulsed-DC electrofishing and gill netting in each reach of the Illinois River that we sampled for Silver Carp density and eDNA. Illinois Natural History Survey (INHS) provided electrofishing data for Starved Rock and Dresden Island reaches as part of the Long Term Survey and Assessment of Large River Fishes in Illinois program [38], and we sampled gillnet catch during removal events conducted by the Illinois Department of Natural Resources (IDNR) in the Dresden Island and Starved Rock reaches [36]. We conducted electrofishing and gillnet sampling in the LaGrange and Alton reaches following the same procedures as INHS and IDNR in the upstream pools. We held fishes in oxygenated water following capture while awaiting measurement, with native fishes immediately released alive after being measured and surviving nonnative fishes euthanized with an overdose of MS-222. All fish captured by both gears were identified to species and measured for total length (mm) and mass (g). Species relative abundances by length class were used later to estimate Silver Carp densities (see “Analysis of hydroacoustic data” section below).

**Fig 2:**
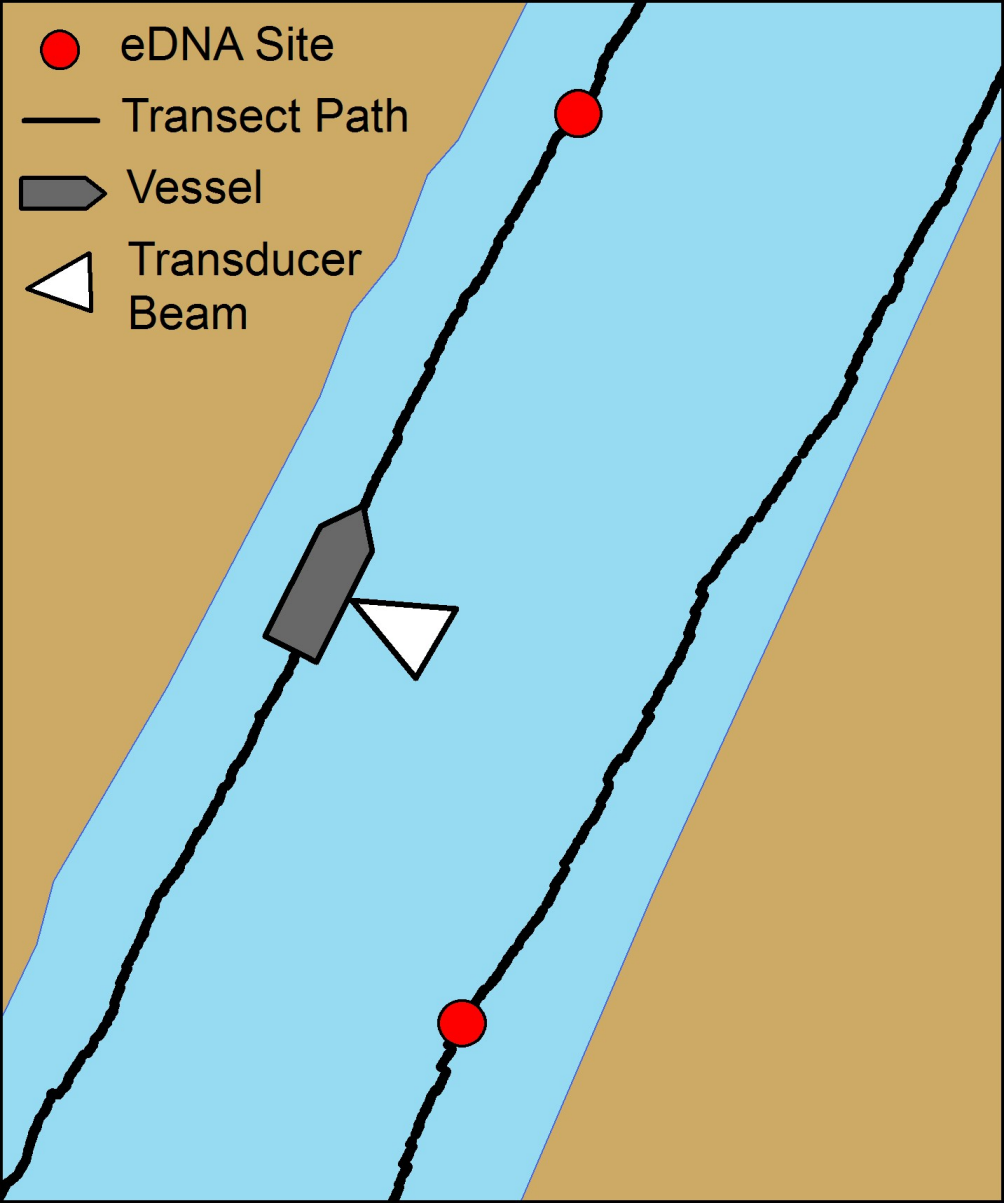
Schematic depicting sampling design for mobile hydroacoustic surveys to assess Silver Carp density and example locations along survey path of surface water sample collection sites (eDNA site) for Silver Carp eDNA analysis. Schematic is not to scale.

We used mobile hydroacoustic surveys to quantify Silver Carp numerical density (individuals per volume of water) and biomass density (mass per volume of water) following established protocols [30,32,36,37]. Hydroacoustic sampling used a 9 m research vessel equipped with two 200-kHz split-beam BioSonics DT-X transducers (BioSonics Inc., Seattle WA, USA). Transducers were horizontally oriented where one transducer was angled near the surface of the water while the second was angled directly below the surface transducer’s beam to maximize volume of water sampled. Both transducers had a ping distance of 50 m, a ping rate of 5 pings·s^-1^, and a 0.4 ms pulse duration. We conducted mobile hydroacoustic surveys by travelling along transect paths parallel to shore at 7.2 km·h^-1^, with transducers beaming away from shore (Fig 2). See [36,37] for a complete description of data collection settings, equipment setup, and sampling design.

### Surface water sample collection for eDNA

We collected surface water samples for eDNA analysis using established protocols that preclude contamination [20]. Briefly, water samples (one sample per location) were taken from the bow of the forward-moving hydroacoustic vessel using 2-L virgin sterile plastic jugs (Uline, Hudson WI) attached to a 3 m long sterilized pole to collect samples several meters in front of the boat (*sensu* [20,21]). Samples were collected approximately 5 cm below the water surface and immediately capped with a virgin cap using gloves and placed on ice. At least one internal negative control jug was collected each day by filling jugs with clean tap water before boarding, carrying them on the boat each day and storing with collected samples in a cooler, opening and recapping onboard, and then returning to the laboratory for extraction. We also collected one water sample at each site for later analysis of water quality, including phosphorus, hardness, total dissolved solids, total suspended solids, and total organic carbon concentrations, in order to characterize conditions at each site. The number of water samples collected at each site for eDNA analysis ranged from 5 to 22 (18–32 total samples per day) and was proportional to the size of each site, with 192 total samples collected throughout the river for the study. We used a stratified random design to determine water sample locations along transect paths (Fig 2) because variability in Silver Carp spatial distributions was unknown. Because Silver Carp were relatively abundant, unfortunately control sites which definitively lacked carp were not available. To accomplish sampling, we divided each site into 0.4 km (non-main channel sites) or 0.8 km (main channel sites) long sections [30,36], and samples were randomly assigned to sections along transect paths (all sections within a site received approximately equal number of samples). When collecting samples for water quality, we also measured water temperature, dissolved oxygen concentration, and specific conductance using a YSI 85 meter (Yellow Springs Instruments, Yellow Springs, OH, USA), and measured Secchi disk depth as an index of water clarity at three locations per site. We recorded GPS coordinates at each sample location.

### eDNA quantification

After being transported from the field, all water samples were stored on ice until filtration which occurred ≤ 24 h after collection per established protocols [20,39]. Water samples (1 L) were filtered following established protocols using glass fiber filters [39,40], a technique that has been shown to extract eDNA more efficiently than other techniques including centrifugation and does not introduce contamination while providing a linear relationship with eDNA concentration [40]. Briefly, after cleaning all equipment and isolated counter tops with a 10% solution of bleach, 1 L of each 2 L sample was passed through Whatman 934-AHTM 1.5 mm glass microfiber filters (GE Whatman, Fairfield, CT, USA) using a polyphenylsulfone filter funnel (Pall Corporation, Port Washington, NY, USA). Filter funnels and forceps were also soaked in 10% bleach and rinsed extensively with distilled water prior to use for each sample. Each filter was stored in a 2 oz Whirl-Pak Write-On Bag (Nasco, Fort Atkinson, WI, USA) and stored on dry ice while in the field and later at -80°C once in the laboratory until DNA extraction occurred. Total DNA was then extracted from filters by using FastDNA Spin Kit (MP Biomedicals, Santa Anna, CA, USA) and further purified through OneStep PCR Inhibitor Removal Kit (Zymo Research, Irvine, CA, USA), with a final DNA elution volume of 50 uL. One laboratory extract control was established for each set of samples by using an unused filter membrane to check for cross-contamination during DNA extraction.

To measure Silver Carp DNA in the extracts, we used an established and frequently used PCR amplification protocol [41] that employs two molecular markers (SC-TM4 and SC-TM5) of Silver Carp mitochondrial DNA. Previous work has demonstrated that these markers are specific to Silver Carp and do not amplify DNA from 29 native fishes in the Illinois River [42] that were also present at our study sites (e.g., common carp (*Cyprinus carpio*) that had high relative abundance; S1 Table). We used two primers to increase our confidence in identifying Silver Carp. Primers SC-TM4_F (5’-CCACTAACATCACCACGCAA-3’), SC-TM4_F (5’-AGCCTTTTCCAGAGGCTTGG-3’) and the probe SC-TM4_P (6-FAM/TAACCCAGC/ZEN/TGCCAATACAA/3IABkFQ) were used for the marker SC-TM4; primers SC-TM5_F (5’-CCACAACTTACCCTCCTTGCC-3’), SC-TM5_F (5’- AAGGGTATTAATTTTTGTGGTGGA-3’), and the probe SC-TM5_P (HEX/TCATGACAT/ZEN/CCGCAGCATTCCTC/IABkFQ) were used for the marker SC-TM5 (Integrated DNA Technologies Inc., Coralville, IA). A synthetic dsDNA fragment (gBlocks Gene Fragments, Integrated DNA Technologies Inc., Coralville, IA) containing the target genes was used for the qPCR standard.

All qPCR reactions were run using established techniques [20,39,40] which were developed to minimize any possibility of contamination (isolated eDNA work spaces; 10% bleach to clean benches, laboratory room, and equipment, including pipettors; setup lab-control samples and filtration equipment; deionized water as the control during the water sample filtration). qPCR reactions were run in 25 μL volumes containing 12.5 μL 2X iTaq Universal Probes Supermix (Bio-Rad, Hercules, CA, USA), 10 mg bovine serum albumin (New England Biolabs Inc., Ipswich, MA, USA), 0.5 μM of each primer, 0.375 μM of each probe, and 5μL of DNA template. Primer and probe sets were run together. Temperature cycling began with an initial denaturation step at 95°C for 3 min, followed by 40 cycles of 95°C for 15 sec and 60°C for 1 min. Each run contained triplicate reactions of no template controls and 30, 300, 3000, 30000, and 300000 copy standards. The assay limit of detection (LOD) was defined as the copy number at which 95% of replicate standard successfully amplify [43], and was determined to be 30 copies·reaction^-1^ based on serial dilution studies conducted in our laboratory using standards as required for linear dynamic range testing procedures [43]. Duplex qPCR were performed using the StepOnePlus Real-Time PCR System (Life Technologies, Grand Island, NY, USA), and Cq values were automatically determined using the system software. Sample marker concentrations were calculated on a per-run basis. The amplification efficiencies from all plates ranged between 96 ‒ 106% for SC-TM4 and 97 ‒ 102% for SC-TM5, which are within normal ranges. We also used serial dilutions of the standard DNA template to check and confirm the LOD of this qPCR protocol. Each qPCR run contained triplicate reactions of standards, non-transcript controls, and samples. We performed qPCR using 1:10 diluted DNA template for all samples to eliminate inhibition which was observed in a few pilot samples using diluted samples. Twenty samples were re-run at different dilutions to confirm results and confirm there was no inhibition. We did not find any cross contamination in any laboratory or field control samples. Concentration of eDNA was averaged across each sample’s replicate reactions prior to data analysis.

### Analysis of hydroacoustic data

We performed post-processing of hydroacoustic data using Echoview 6.1 software (Echoview Software Pty Ltd, Hobart, Tasmania, Australia). We established fixed nearfield exclusion lines 1 m away from both transducers and manually drew the bottom exclusion lines where each transducer’s acoustic beam intersected the river bed. Thus, fish targets in between the nearfield and bottom exclusion lines were included in analyses. First, we filtered background noise using a -60 dB threshold and then identified acoustic targets using Echoview’s ‘fish track detection’ algorithm. We then manually inspected and edited individual fish tracks and calculated fish length from target strength using the side aspect equation from [44]. Volume of water ensonified between the nearfield and bottom exclusion lines was determined using Echoview’s ‘wedge volume sampled’ method. Detailed descriptions of all post-processing procedures are described in [36,37].

Silver Carp densities were estimated following procedures described elsewhere [30,36,37]. Briefly, hydroacoustic survey paths were separated into spatial intervals (0.925 km long for main channel sites; 0.463 km long for non-main channel sites) for which densities were separately calculated. Reach-specific proportion of catch data comprised of Silver Carp was calculated for each 0.1 cm length increment (electrofishing and netting catch data combined to minimize gear bias in species composition; S1 Table). The proportion of Silver Carp by length increment was then applied to the observed number of hydroacoustic fish targets in each length increment to estimate number of Silver Carp per increment (e.g., a length bin comprised of 50% Silver Carp from catch data and 20 hydroacoustic fish targets results in an estimate of 10 Silver Carp). Silver Carp biomass was then estimated using the reach-specific length-mass relationship for Silver Carp from the catch data. Summing number and biomass across length increments provided both the estimated total number and mass of Silver Carp. This was converted to numerical density and biomass density within each interval by dividing the number or mass of Silver Carp by the water volume sampled in each interval. We calculated numerical density and biomass density and related both indices to eDNA because size distributions of Silver Carp vary across river reaches in our study system [30] which could affect eDNA (e.g., similar number of fish between two sites but different total biomass; *sensu* [15,16]).

### Analyzing relationships between eDNA and Silver Carp density

We used the GPS coordinates for each eDNA water sample to identify the corresponding density interval to match each sample’s eDNA concentration with Silver Carp density on a relatively fine spatial scale. Silver Carp density at a site was then calculated as the mean density of the intervals where eDNA sampling occurred. This procedure was conducted (as opposed to using densities from all transect intervals) to match Silver Carp density near eDNA samples.

After attempting several types of linear regression which provided poor fits, we modeled relationships between Silver Carp densities and eDNA concentration using nonlinear regression (‘nls’ function in the ‘nlstools’ package in R) following the Michaelis-Menten equation:

$$eDNA=\frac{V(Density)}{K(Density)},$$ (1)

where eDNA is eDNA concentration (copies 100 mL^-1^), density is numerical (number 1000 m^-3^) or biomass density (kg 1000 m^-3^), and *V* and *K* are estimated parameters. This equation fit the observed relationship well and is commonly used in nonlinear analysis. Starting values were set as half of the maximum observed eDNA concentration for parameter *K*, and the maximum observed eDNA concentration for parameter *V*. Models were fit using bootstrap resampling (10000 resamples) to account for observed variability in eDNA concentration and density at each site, with final parameter estimates calculated as the median of resampled estimates. Residual plots were generated and inspected (S1 Fig), and bootstrapped 95% confidence intervals were calculated using the ‘predictNLS’ function in R (‘propagate’ package). We used generalized linear models to assess the relationships between Silver Carp densities and eDNA detection rate at a site (percent of samples with eDNA concentration > LOD; ‘glm’ function in program R 3.0.2; binomial family, logit link). These models were also bootstrap resampled 10000 times to incorporate observed variability in density at each site. Water quality measurements were not included as covariates in analyses because these measurements were collected at a broad spatial scale (e.g., 1 or 3 samples per site regardless of site size) which likely did not reflect variability throughout the entire site, particularly when attempting to match with fine-scale eDNA and density measurements. Instead, water quality data reflect a broad characterization of site conditions and are reported for reference (S2 Table).

## Results

Silver Carp density varied among sites but was generally higher in Alton and LaGrange reaches and lower in Dresden Reach. Numerical density ranged from 0.0 ‒ 14.6 Silver Carp·1000 m^-3^ and biomass density ranged from 0.0 ‒ 18.8 kg·1000 m^-3^. Water quality varied throughout the river, with reaches in the upper river (Dresden Island and Starved Rock reaches) generally having high water clarity (higher Secchi depth) relative to reaches in the lower river (Alton and LaGrange reaches; S2 Table). However, water quality variables, including water temperature, displayed similar among-site variation across river reaches.

All 8 negative field control samples, 14 laboratory extraction negative controls, and template reaction negative controls had concentrations below LOD for both primers. Results from the qPCR of SC-TM4 were very similar to SC-TM5 so only SC-TM4 results are included here (see supporting information for SC-TM5 results). The concentration of eDNA ranged from < LOD to 52909 copies·L^-1^, with detection rate varying throughout the river between 38% ‒ 100%.

**Fig 3:**
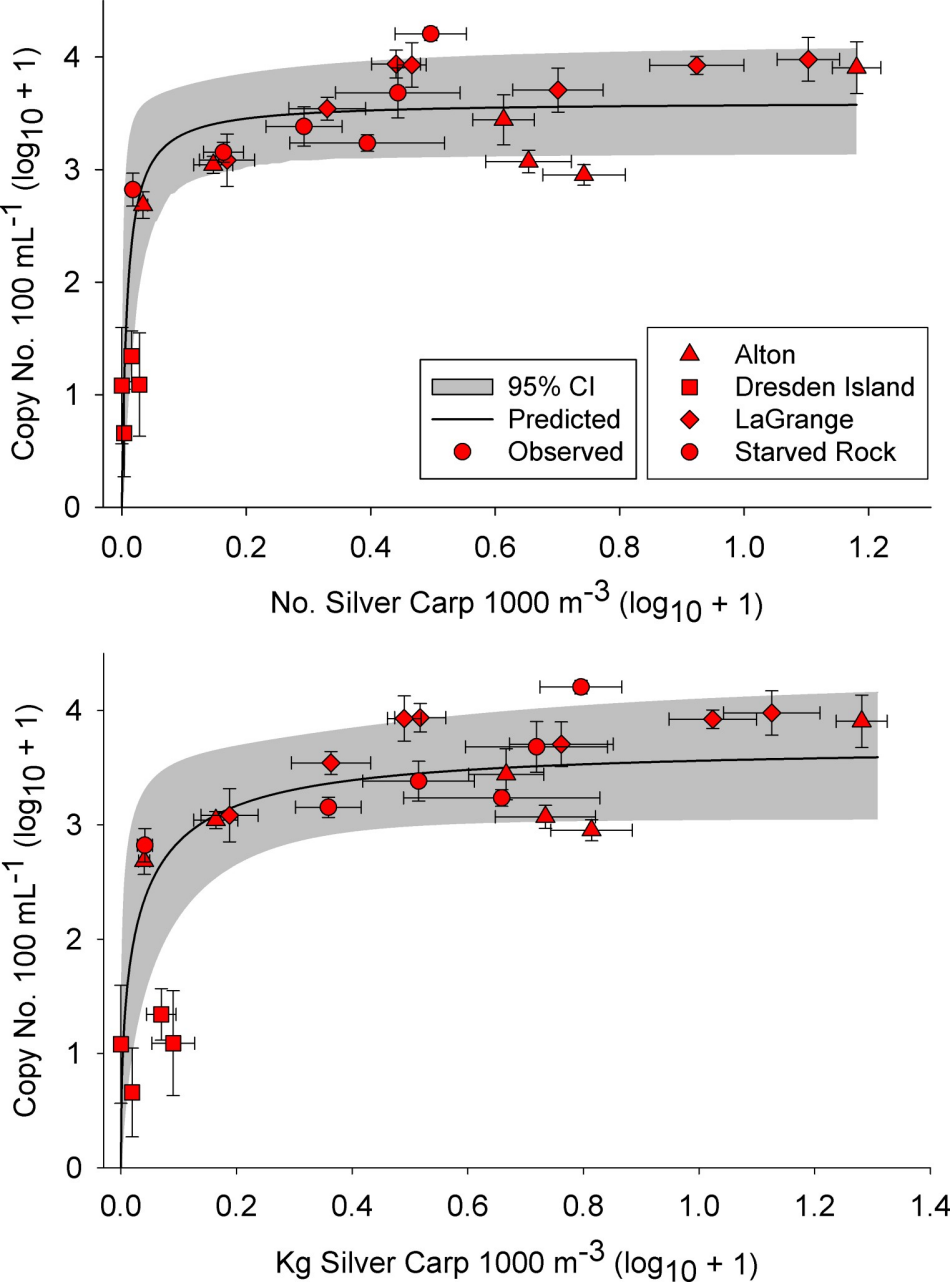
Relationships between site mean (standard error; SE) Silver Carp density and mean (SE) eDNA concentration (SC-TM4 marker) collected from the Illinois River, USA. Symbols represent river reach (see Fig 1) and error bars reflect variability among samples at a site.

**Fig 4:**
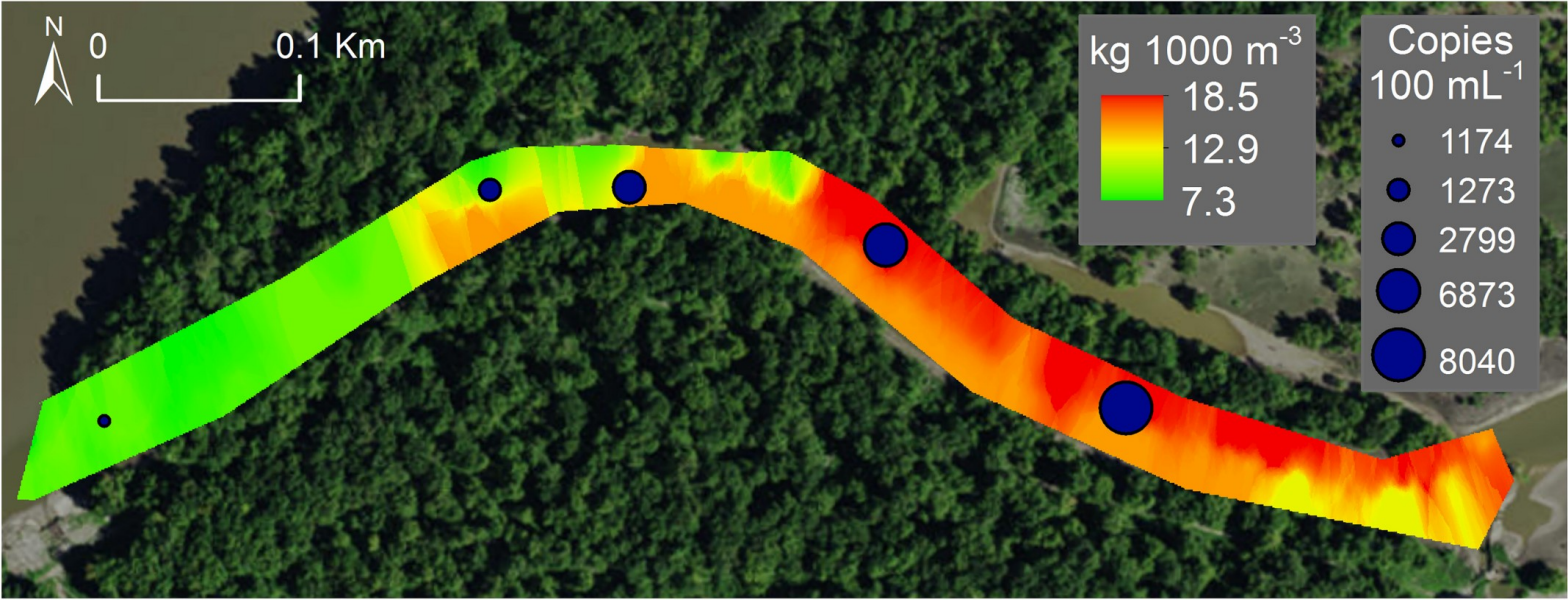
Example of spatial relationships within a site between Silver Carp biomass density (kg·1000 m^-3^) determined from mobile hydroacoustic sampling and eDNA concentration (SC-TM4 marker) from surface water at a side channel habitat in the LaGrange Reach of the Illinois River, USA.

**Fig 5:**
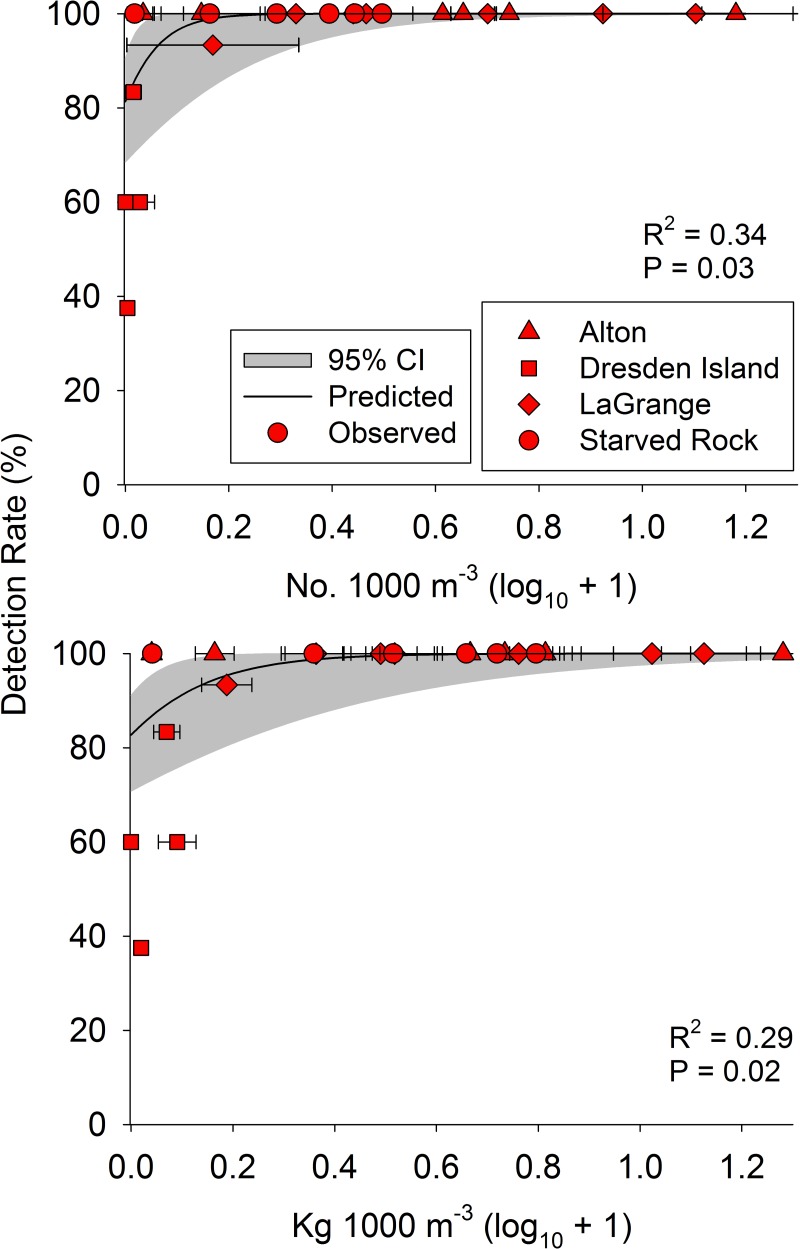
Relationships between site mean (standard error) Silver Carp density and percent of samples with detectable eDNA concentrations (Detection Rate; SC-TM4 marker; R^2^: McFadden’s R^2^) collected from the Illinois River, USA. Symbols represent river reach (see Fig 1) and error bars reflect variability among samples at a site.

Silver carp eDNA concentration (copy number) was nonlinearly related to both Silver Carp numerical density (*K* = 0.008 ±0.003 SE; *V* = 3.60 ±0.03; RMSE = 0.84) and biomass density (*K* = 0.009 ±0.0007 SE; *V* = 3.55 ±0.006; RMSE = 0.89). Concentration of eDNA rapidly increased at low Silver Carp densities and plateaued at intermediate and high densities of Silver Carp (Fig 3 and S2 Fig). Copy number also qualitatively displayed nonlinear relationships with Silver Carp densities within sites (Fig 4). We also examined relationships between density and eDNA concentration excluding samples with undetectable levels of eDNA and found similar nonlinear relationships (S3 Fig). Silver Carp detection rate was related to both Silver Carp numerical density (McFadden’s R^2^ = 0.34, z = 2.2, df = 21, P = 0.03) and biomass density (McFadden’s R^2^ = 0.29, z = 2.3, df = 21, P = 0.02; Fig 5 and S4 Fig). Sites with more than 0.08 Silver Carp·1000 m^-3^ (0.10 kg·1000 m^-3^) had 100% detection rates except for one location in the main channel of the LaGrange reach. This site (MC2 in S2 Table) had 93% detection rate (N = 15 samples) and densities of 0.59 Silver Carp·1000 m^-3^ and 0.69 kg·1000 m^-3^. This site also had the highest concentration of total suspended solids and low water clarity (low Secchi depth; S2 Table).

## Discussion

This study, one of only a few to quantify the relationship between the density of a fish and its eDNA concentration in a large river, identifies a significant nonlinear relationship between Silver Carp density and the eDNA they release in the Illinois River. While previous studies [1,12,45] have shown that detection of Silver Carp eDNA can accurately predict the presence of this species in rivers, our study both confirms these results and, for the first time, shows there is a quantitative relationship between the density of Silver Carp and the quantity of their DNA in natural waters. These results indicate that quantification of eDNA concentration is a promising tool for population assessments and management programs in rivers for this important invasive species, and perhaps others, especially at low densities in newly invaded areas.

We describe a nonlinear relationship between eDNA concentration and Silver carp density that can be described by a Michaelis-Menten relationship which rapidly increases in eDNA concentration at low densities of Silver Carp and quickly plateaus. A similar relationship was observed between the density of ayu (*Plecoglossus altivelis*) and their eDNA in small Japanese streams which varied with season, with the greatest nonlinearity occurring in mid-summer; however, saturation was not observed [23]. Similarly, Ghosal et al. [21], found a nonlinear relationship between the density of aggregating common carp and eDNA concentration in a moderate-sized lake in late summer while noting that feeding activity drove large increases in eDNA release which decreased rapidly within a few meters. In contrast, Lacoursière‐Roussel et al. [13] found a nearly linear relationship between lake trout (*Salvelinius namaycush*) abundance and eDNA in the early spring in 12 lakes. Linear relationships between eDNA release and fish abundance have also been consistently measured in small aquaria and ponds, including for various carp species, including Silver Carp [16,17]. However, small aquaria and ponds do not suffer from issues of dilution, sampling error, and complex water chemistry which is associated with eDNA binding, inhibition, and perhaps decay [15]. Together these relationships suggest that many factors drive the fish density-eDNA relationship in natural water but which almost certainly includes both eDNA production/release as well as decay.

The causes of the nonlinear relationship, which included apparent saturation between Silver Carp density and their eDNA, are unknown but previous work hints at possible mechanisms. Nonlinear relationships between eDNA-and fish density have previously been observed at warmer seasons [23] similar to conditions during this study (~ 20°C). Temperature would have both direct effects, via increased eDNA decay rates, and indirect effects by influencing feeding rates and mucous (eDNA) sloughing. Of course, fish were also likely actively moving at the time of our study, which may have reduced precision and contributed to apparent saturation. We strongly suspect eDNA decay was a very important factor as seen by Ghosal et al. [21] with Common Carp. Likely eDNA values did not go to zero in our study because of water flowing into all sampling regions from upstream regions which contained Silver Carp (infestation levels are high in the Illinois River). Notably, we did not measure inhibition in our analyses and none of our many control measurements measured eDNA, so neither water chemistry nor contamination is an explanation for the relationships we described. Probe specificity can also be ruled out as both probes, which were extensively evaluated for specificity, showed very similar results. It is very possible that sampling Silver Carp and their eDNA at different times of the year may produce different relationships as seen by Doi et al. [23]. Illinois River water presumably also had higher organic loads than Japanese streams which may lead to higher binding at higher eDNA concentrations than noted by Doi et al. [23]. The nature and causes of the relationship between Silver Carp density and eDNA in rivers strongly warrants systematic study.

Our discovery of a quantifiable nonlinear relationship between Silver Carp density and their eDNA suggests that eDNA concentration can be of great value to Silver Carp and invasive fish management in large rivers. Notably, our work also supports findings from previous studies [12,45,46] that eDNA accurately indicates the presence of invasive riverine fish and, thus, can be reliably used for surveillance (e.g., detection rate data). Additionally, we now show that low concentrations of eDNA reflect, and can be used to estimate, low densities of Silver Carp which would be extremely useful for determining relative Silver Carp abundances in newly invaded locations or where low densities otherwise exist. While the trends we observed do not allow us to use eDNA concentration to discern between moderate and high Silver Carp densities, water samples containing intermediate or high eDNA concentration still provides managers with an estimate of a minimum threshold Silver Carp density (i.e., the density at which the eDNA concentration-density curves plateau). Of course, different, possibly linear relationships may exist at different times of the year but this has yet to be determined. Future research will hopefully identify the mechanisms underlying these relationships.

In addition to using well established procedures to measure eDNA and employing a large sampling grid, another important reason we were able to identify relationships between eDNA and density was likely our use of mobile hydroacoustic sampling to assess the fish population. This approach allowed us to rapidly and accurately sample a large amount of the river and across a variety of habitats. Other studies have used similar approaches for relating fish abundance to eDNA but not in a river. Yamamoto et al. [14] used a hydroacoustic approach to quantify fish biomass and successfully determine relationships with eDNA concentration in a marine environment. In contrast, most field studies have used traditional capture gears and had mixed results when relating eDNA concentration to indices of abundance or biomass [13,18,19,22]. As with any gear, hydroacoustic sampling has limitations and bias (e.g., as outlined in [36,37]) that can affect density estimates and relationships with eDNA. For example, hydroacoustic sampling is not well suited for use in very shallow (e.g., < 1 m deep) habitats or for benthic species. Careful consideration of habitat characteristics and species behavior are essential when selecting sampling gears when attempting to relate indices of abundance to eDNA [19]. We suggest future studies evaluating relationships between abundance or biomass and eDNA consider mobile hydroacoustic surveys as an approach for surveying non-benthic fish populations, particularly in large systems with diverse habitats.

The challenges posed by large river fisheries require novel assessment techniques. In this study, we demonstrated how a non-traditional gear for indexing riverine fish abundance was related to eDNA in ways that can inform the management of invasive Silver Carp by providing managers with relative abundance estimates relative to both observed eDNA detection rate and concentration. Future work should evaluate the sensitivity of eDNA as an indicator of presence and abundance in rivers by identifying the density threshold above which water samples at field sites yield detectable eDNA concentrations at different times of the year. Improvements are also needed in understanding processes affecting the fate and transport of eDNA in rivers. Identifying relationships between fish density and eDNA will benefit aquatic species monitoring and management programs, especially for invasive or endangered species, for which density estimates are also needed.

## Supporting information

S1 Table Species composition from fish capture data. (DOCX)

S2 Table Summarized eDNA detection rates and water quality data. (DOCX)

S1 Fig Residual plots of nonlinear regressions modeling relationships between Silver Carp density (numerical or biomass) and eDNA concentration (markers SC-TM4 and SC-TM5). (TIF)

S2 Fig Relationships between SC-TM5 eDNA concentration and density. Symbols represent river reach (see Fig 1) and error bars reflect variability among samples at a site.
(TIF)

S3 Fig Relationships between SC-TM4 eDNA concentration and density excluding samples with undetectable levels of eDNA. Symbols represent river reach (see Fig 1) and error bars reflect variability among samples at a site.
(TIF)

S4 Fig Relationships between SC-TM5 eDNA detection rate and density. Symbols represent river reach (see Fig 1) and error bars reflect variability among samples at a site.
(TIF)

S1 Dataset Silver Carp densities and eDNA concentrations from study sites collected throughout the Illinois River. (XLSX)
