# Supplementary material for: Nonlinear relationship between Silver Carp density and their eDNA concentration in a large river
Source: PLoS One. 2019 Jun 26;14(6):e0218823. doi: 10.1371/journal.pone.0218823 (PMC6594630; doi:10.1371/journal.pone.0218823)
Supplement: S1 Table — (DOCX) [file pone.0218823.s001.docx]

**Coulter et al. Silver Carp density relates nonlinearly with their eDNA concentration in a large river**

**S1 Table.** **Species composition from fish capture data.** Relative abundance (percent of catch) of fish species from capture sampling in river reaches where Silver Carp densities and eDNA were also sampled in autumn 2016 in the Illinois River, USA.

|  |  | **Relative Abundance** | | |
| --- | --- | --- | --- | --- |
| **Pool** | **Species** | **Electrofishing** |  | **Gillnet** |
| Dresden | Bighead Carp |  |  | 3.7 |
|  | Bigmouth Buffalo |  |  | 3.7 |
|  | Black Buffalo |  |  | 3.0 |
|  | Bluegill | 11.0 |  |  |
|  | Bluntnose Minnow | 2.4 |  |  |
|  | Channel Catfish | 0.6 |  | 4.4 |
|  | Common Carp | 6.1 |  | 40.0 |
|  | Flathead Catfish |  |  | 0.4 |
|  | Freshwater Drum | 3.0 |  | 1.1 |
|  | Gizzard Shad | 58.5 |  |  |
|  | Goldfish |  |  | 1.1 |
|  | Golden Shiner | 1.8 |  |  |
|  | Green Sunfish | 1.2 |  |  |
|  | Largemouth Bass | 5.5 |  |  |
|  | Longnose Gar | 0.6 |  | 1.5 |
|  | River Carpsucker |  |  | 0.4 |
|  | Rock Bass | 1.8 |  |  |
|  | Silver Carp |  |  | 4.4 |
|  | Smallmouth Bass | 3.7 |  |  |
|  | Smallmouth Buffalo | 2.4 |  | 36.7 |
|  | Spottail Shiner | 0.6 |  |  |
|  | White Sucker | 0.6 |  |  |
| Starved Rock | Banded Killifish | 0.3 |  |  |
|  | Bighead Carp |  |  | 9.3 |
|  | Black Crappie | 0.8 |  |  |
|  | Bluegill | 4.1 |  |  |
|  | Bigmouth Buffalo |  |  | 0.3 |
|  | Bullhead Minnow | 4.1 |  |  |
|  | Channel Catfish | 1.4 |  |  |
|  | Channel Shiner | 0.8 |  |  |
|  | Common Carp | 1.4 |  | 0.7 |
|  | Emerald Shiner | 8.9 |  |  |
|  | Freshwater Drum | 1.9 |  | 0.3 |
|  | Gizzard Shad | 37.9 |  |  |
|  | Golden Redhorse | 1.1 |  |  |
|  | Grass Carp | 0.5 |  |  |
|  | Green Sunfish | 0.8 |  |  |
|  | Johnny Darter | 0.3 |  |  |
|  | Largemouth Bass | 3.3 |  |  |
|  | Logperch | 2.2 |  |  |
|  | Longnose Gar | 0.5 |  |  |
|  | Northern Hogsucker | 0.3 |  |  |
|  | Orange Spotted Sunfish | 1.9 |  |  |
|  | River Carpsucker | 1.1 |  |  |
|  | River Shiner | 1.6 |  |  |
|  | Sauger |  |  | 0.3 |
|  | Shorthead Redhorse | 0.8 |  |  |
|  | Shortnose Gar | 0.3 |  |  |
|  | Silver Carp | 1.6 |  | 86.9 |
|  | Slenderhead Darter | 0.3 |  |  |
|  | Smallmouth Bass | 4.9 |  |  |
|  | Smallmouth Buffalo | 10.6 |  | 2.1 |
|  | Spotfin Shiner | 3.0 |  |  |
|  | Spottail Shiner | 3.0 |  |  |
|  | White Perch | 0.5 |  |  |
| LaGrange | Bighead Carp |  |  | 5.5 |
|  | Black Buffalo | 0.2 |  |  |
|  | Black Carp | 0.2 |  |  |
|  | Bluegill | 0.4 |  |  |
|  | Bigmouth Buffalo | 0.2 |  | 9.2 |
|  | Bowfin | 0.2 |  |  |
|  | Common Carp | 2.8 |  | 3.7 |
|  | Emerald Shiner | 6.5 |  |  |
|  | Flathead Catfish | 0.4 |  | 0.9 |
|  | Freshwater Drum | 0.4 |  | 2.8 |
|  | Goldfish | 0.2 |  |  |
|  | Green Sunfish | 0.2 |  |  |
|  | Grass Carp | 1.1 |  | 5.5 |
|  | Gizzard Shad | 19.5 |  |  |
|  | Highfin Carpsucker | 0.4 |  |  |
|  | Longnose Gar | 0.7 |  |  |
|  | Quillback | 0.2 |  |  |
|  | River Redhorse | 0.2 |  |  |
|  | Sauger | 0.7 |  |  |
|  | Skipjack Herring | 0.4 |  |  |
|  | Sliver Carp | 61.0 |  | 65.1 |
|  | Smallmouth Buffalo | 1.1 |  | 7.3 |
|  | Shortnose Gar | 0.4 |  |  |
|  | Silver Redhorse | 0.2 |  |  |
|  | Walleye | 0.2 |  |  |
|  | White Bass | 1.7 |  |  |
|  | White Crappie | 0.2 |  |  |
| Alton | Black Buffalo | 0.4 |  |  |
|  | Bluegill | 0.8 |  |  |
|  | Bigmouth buffalo | 2.9 |  | 6.3 |
|  | Common Carp | 5.8 |  | 17.5 |
|  | Channel Catfish | 1.3 |  |  |
|  | Emerald Shiner | 3.8 |  |  |
|  | Freshwater Drum | 0.4 |  | 1.6 |
|  | Grass Carp | 0.8 |  | 31.7 |
|  | Gizzard Shad | 14.2 |  |  |
|  | Largemouth Bass | 0.4 |  |  |
|  | Longnose Gar | 2.9 |  |  |
|  | Mooneye | 0.4 |  |  |
|  | Skipjack Herring | 1.3 |  |  |
|  | Sliver Carp | 53.8 |  | 33.3 |
|  | Smallmouth Buffalo | 1.7 |  | 9.5 |
|  | Shortnose Gar | 3.8 |  |  |
|  | White Bass | 5.4 |  |  |
