# Supplementary material for: Nonlinear relationship between Silver Carp density and their eDNA concentration in a large river
Source: PLoS One. 2019 Jun 26;14(6):e0218823. doi: 10.1371/journal.pone.0218823 (PMC6594630; doi:10.1371/journal.pone.0218823)
Supplement: S2 Table — (DOCX) [file pone.0218823.s002.docx]

**Coulter et al. Silver Carp density relates nonlinearly with their eDNA concentration in a large river**

S2 Table. Detection rates of Silver Carp eDNA (SC-TM4 and SC-TM5 markers) and water quality measurements at each site. Samples were collected across four reaches of the Illinois River, USA during October 2016 in main channel (MC), backwater (BW), side channel (SC), and tributary (T) habitats. Phos: total phosphorus; Hard: calcium carbonate hardness; TSS: total suspended solids; Temp: water temperature; DO: dissolved oxygen; Cond: conductivity; Secchi: Secchi disk depth. Several water quality measurements for one site were not collected (NA= not available).

| Reach | Site | Habitat |  | SC-  TM4 | SC-  TM5 | Phos  mg/L | Hard  mg/L | TSS  mg/L | TOC  mg/L | Temp  °C | DO  mg/L | Cond  µS/cm | Secchi  cm |
| --- | --- | --- | --- | --- | --- | --- | --- | --- | --- | --- | --- | --- | --- |
| Dresden | Mobil | BW |  | 60 | 60 | 0.68 | 219 | 12 | 5.0 | 19.3 | 8.5 | 787 | 134 |
|  | MC | MC |  | 83 | 92 | 0.72 | 219 | 23 | 5.2 | 19.5 | 8.5 | 835 | 80 |
|  | Treats | SC |  | 60 | 60 | 0.57 | 231 | 16 | 5.0 | 18.2 | 7.2 | 763 | 68 |
|  | Kankakee | T |  | 38 | 50 | 0.05 | 353 | 10 | 4.5 | 15.3 | 9.4 | 564 | 98 |
| Starved Rock | Heritage | BW |  | 100 | 100 | 0.27 | 289 | 24 | 5.2 | 16.6 | 9.1 | 720 | 37 |
|  | MC | MC |  | 100 | 100 | 0.20 | 308 | 18 | 4.8 | 17.2 | 9.9 | 760 | 57 |
|  | Bulls | SC |  | 100 | 100 | 0.25 | 316 | 14 | 4.6 | 16.1 | 10.0 | 768 | 68 |
|  | Hitt | SC |  | 100 | 100 | 0.21 | 317 | 19 | 4.6 | 16.2 | 9.9 | 773 | 60 |
|  | Sheehan | SC |  | 100 | 100 | 0.24 | 320 | 15 | 4.6 | 16.1 | 9.7 | 791 | 69 |
|  | Fox | T |  | 100 | 100 | 0.23 | 353 | 15 | 5.4 | 13.9 | 10.8 | 734 | 61 |
| LaGrange | MC 1 | MC |  | 100 | 100 | NA | NA | NA | NA | 19.7 | 8.0 | 716 | 24 |
|  | MC 2 | MC |  | 93 | 93 | 0.29 | 310 | 262 | 4.5 | 19.2 | 7.9 | 709 | 23 |
|  | Bath | SC |  | 100 | 100 | 0.30 | 311 | 70 | 4.5 | 19.3 | 7.8 | 715 | 20 |
|  | Chain Lake | SC |  | 100 | 100 | 0.41 | 295 | 54 | 7.1 | 18.5 | 7.7 | 675 | 16 |
|  | Lilly | SC |  | 100 | 100 | 0.52 | 321 | 20 | 4.2 | 19.6 | 7.3 | 739 | 23 |
|  | Quiver | SC |  | 100 | 100 | 0.30 | 317 | 63 | 4.3 | 19.2 | 7.9 | 725 | 20 |
|  | Spoon | T |  | 100 | 100 | 0.18 | 398 | 64 | 2.8 | 17.6 | 8.2 | 766 | 22 |
| Alton | MC | MC |  | 100 | 100 | 0.30 | 304 | 82 | 4.6 | 18.5 | 8.4 | 701 | 37 |
|  | Big Blue | SC |  | 100 | 100 | 0.35 | 307 | 129 | 4.3 | 17.7 | 9.0 | 745 | 43 |
|  | Buckhorn | SC |  | 100 | 100 | 0.34 | 304 | 65 | 4.4 | 18.7 | 8.5 | 700 | 22 |
|  | Dark | SC |  | 100 | 100 | 0.33 | 307 | 46 | 4.4 | 17.4 | 9.1 | 752 | 53 |
|  | Macoupin | SC |  | 100 | 100 | 0.10 | 305 | 15 | 3.8 | 17.5 | 9.2 | 704 | 58 |
|  | McEvers | SC |  | 100 | 100 | 0.31 | 311 | 113 | 4.2 | 18.5 | 7.9 | 701 | 30 |
