# Supplementary figures and images for: Nonlinear relationship between Silver Carp density and their eDNA concentration in a large river
Source: PLoS One. 2019 Jun 26;14(6):e0218823. doi: 10.1371/journal.pone.0218823 (PMC6594630; doi:10.1371/journal.pone.0218823)

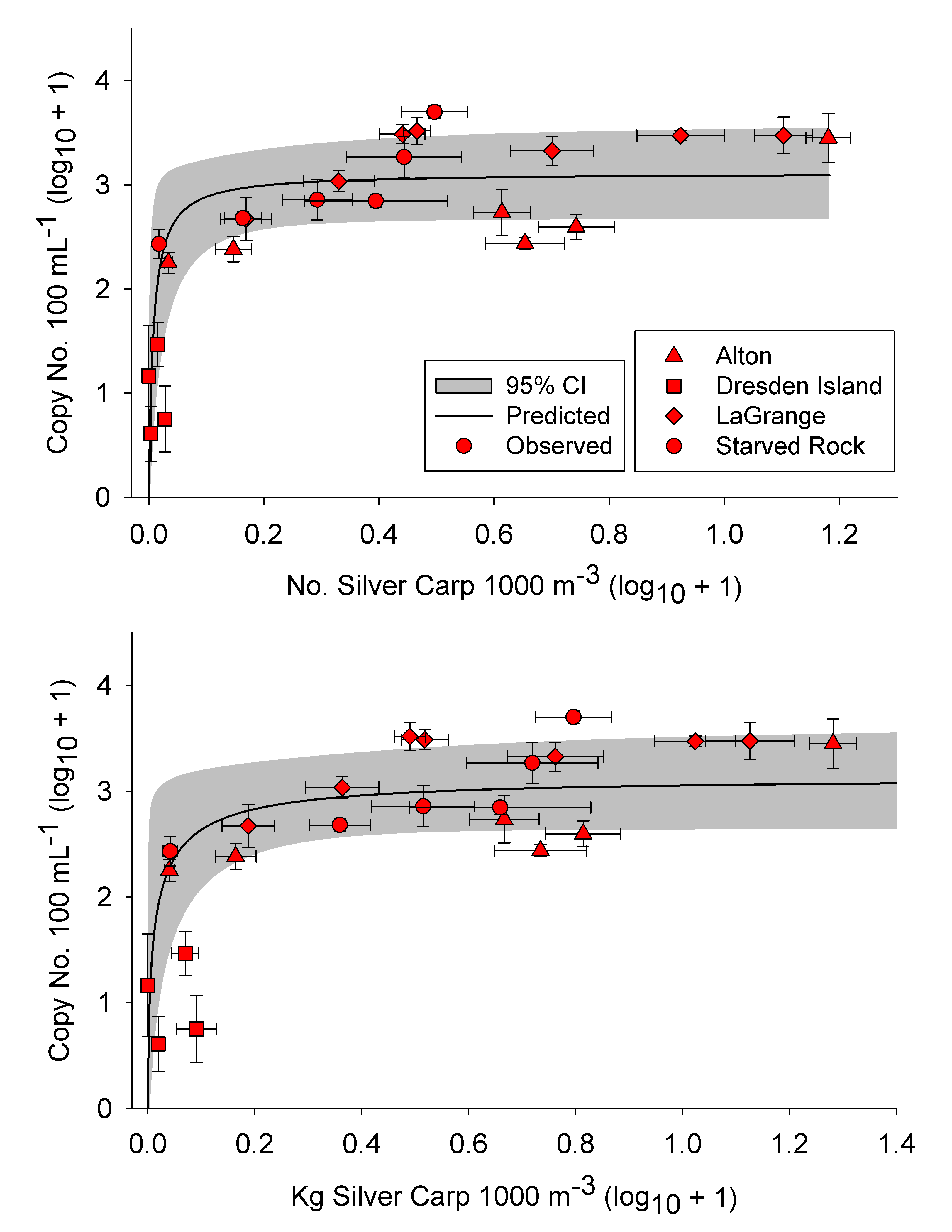

Supplement: S2 Fig — Symbols represent river reach (see Fig 1) and error bars reflect variability among samples at a site. (TIF) [file pone.0218823.s004.tif]

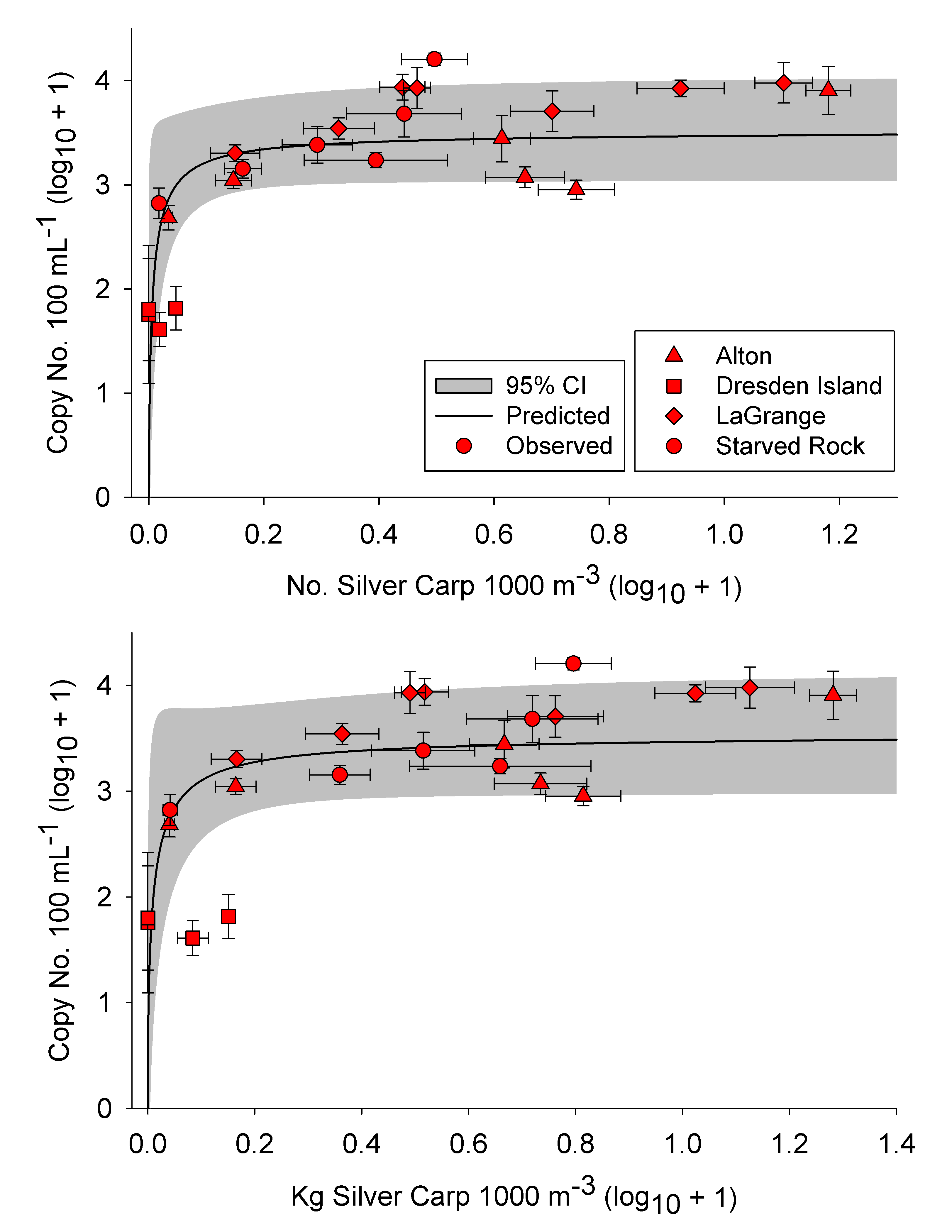

Supplement: S3 Fig — Symbols represent river reach (see Fig 1) and error bars reflect variability among samples at a site. (TIF) [file pone.0218823.s005.tif]

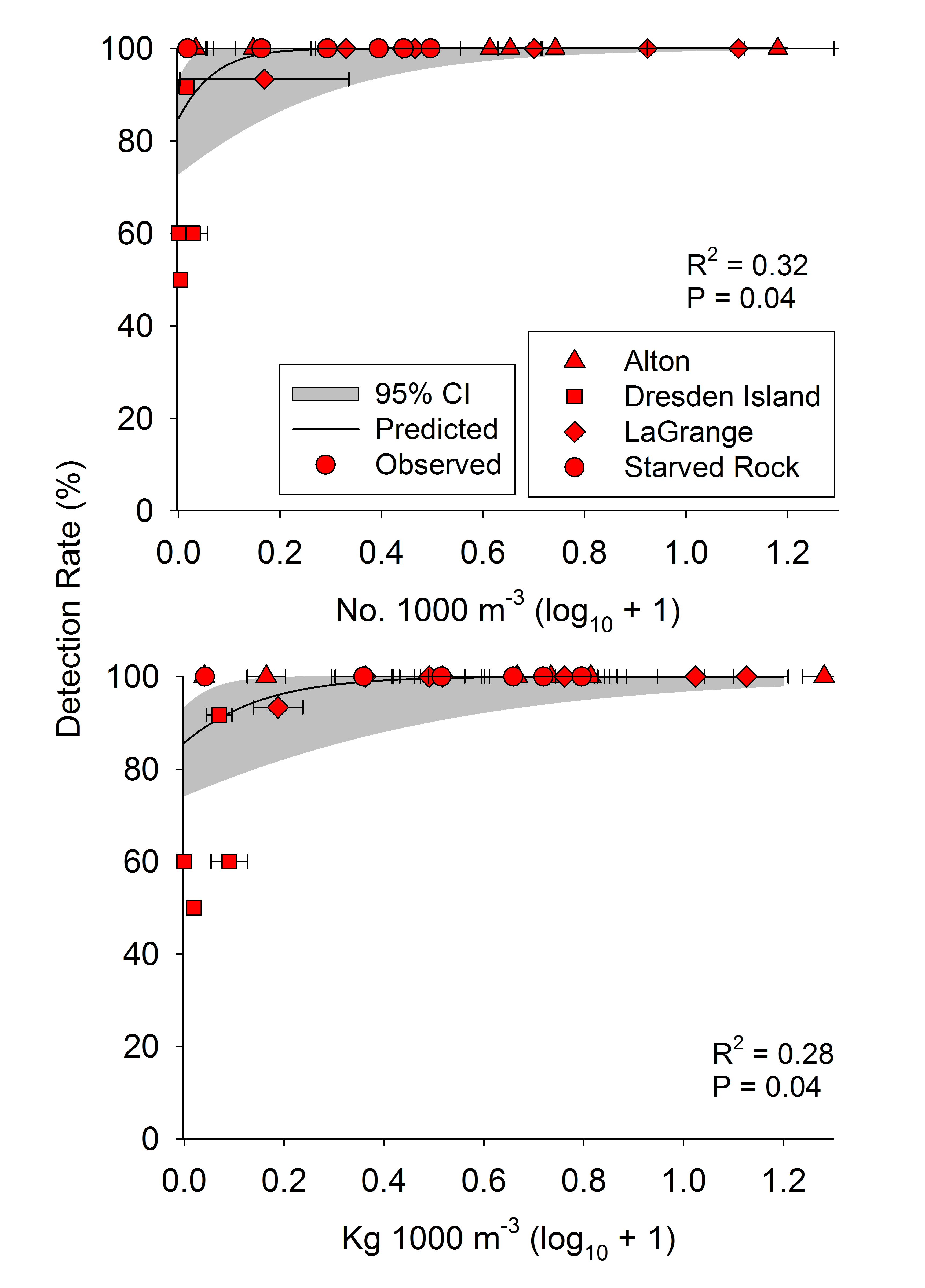

Supplement: S4 Fig — Symbols represent river reach (see Fig 1) and error bars reflect variability among samples at a site. (TIF) [file pone.0218823.s006.tif]
